# Supplementary material for: Historical Silk: A Novel Method to Evaluate Degumming with Non-Invasive Infrared Spectroscopy and Spectral Deconvolution
Source: Materials (Basel). 2023 Feb 22;16(5):1819. doi: 10.3390/ma16051819 (PMC10003773; doi:10.3390/ma16051819)
Supplement: Supplementary file 1 [file materials-16-01819-s001.zip › materials-2168612-supplementary.pdf]

Figure S1. Scheme of the main parts of the traditional Japanese armour.

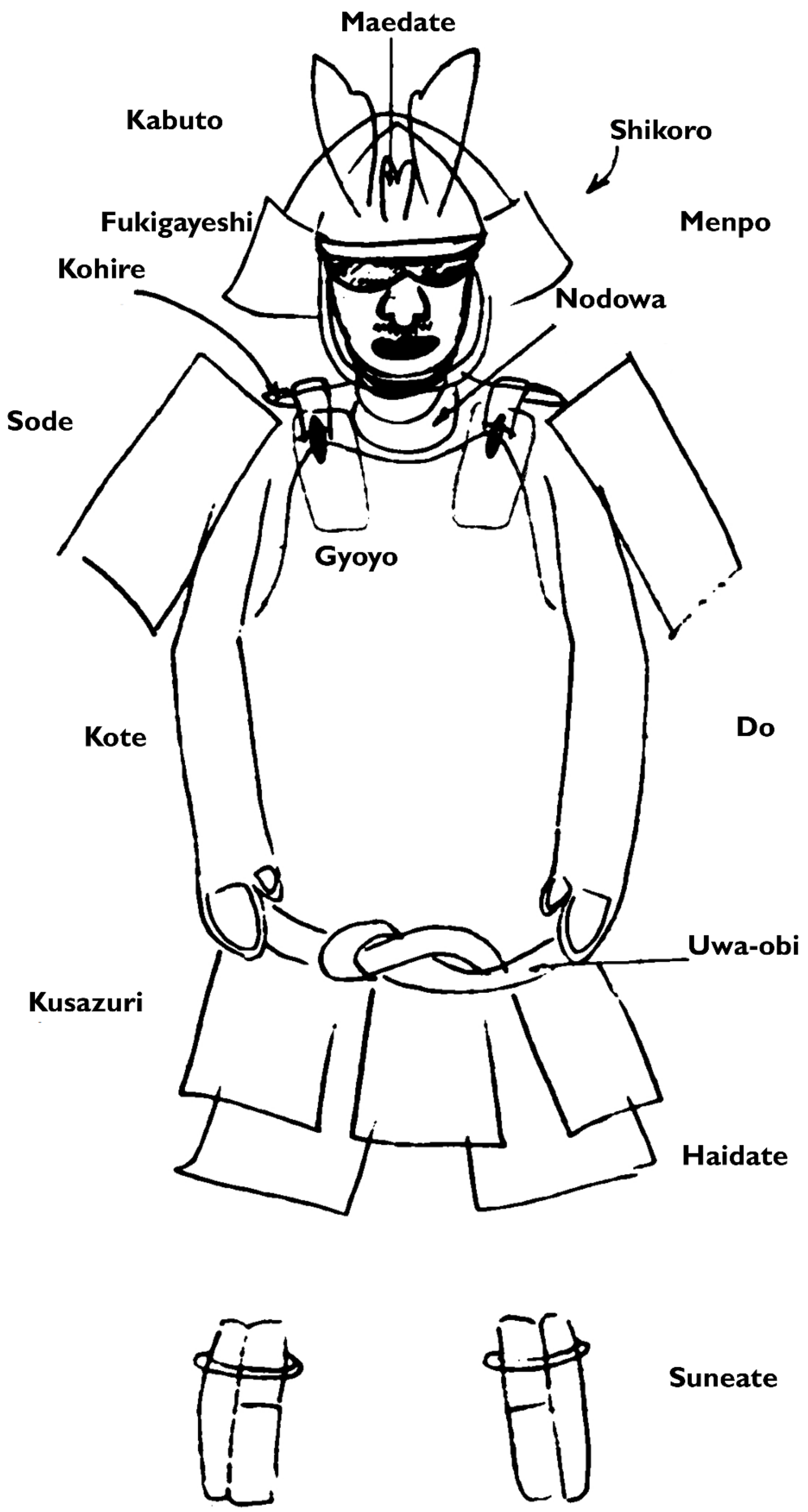

Table S1. List of samples from the Morigi collection. Each sample is linked to its armour reported with its inventory number. On the right, for each sample the presumed dating, the colour, the armour part which the sample comes from and the kind of textile are reported.

Sample	Armour	Presumed dating	Colour	Armour part	Kind of textile
1_2	2017.Mor.1	late 16th c.	orange	kabuto	chin cord
1_6	2017.Mor.1	late 16th c.	orange	nodowa	lacing braid
1_8	2017.Mor.1	late 16th c.	white	sode	lacing braid
1_9	2017.Mor.1	late 16th c.	orange	sode	lacing braid
1_26	2017.Mor.1	late 16th c.	blue	suneate	lacing braid
2_2	2017.Mor.2	late 19th c.	orange	nodowa	lacing braid
2_3	2017.Mor.2	late 19th c.	orange	shikoro	lacing braid
3_3	2017.Mor.3	17th c.	white	kusazuri	lacing braid
3_4	2017.Mor.3	17th c.	green	haidate	embroidered lining
3_8	2017.Mor.3	17th c.	green	kusazuri	lacing braid
3_10	2017.Mor.3	17th c.	blue	do	cord
3_11	2017.Mor.3	17th c.	orange	sode	lacing braid
3_12	2017.Mor.3	17th c.	blue	sode	lacing braid
3_18	2017.Mor.3	17th c.	blue	casacca	brocade lining
3_23	2017.Mor.3	late 16th c.	orange	kabuto	chin cord
4_2	2017.Mor.4	18th c.	blue	haidate	brocade lining
4_3	2017.Mor.4	18th c.	blue	kusazuri	lacing braid
4_4	2017.Mor.4	18th c.	light orange	kusazuri	lacing braid
4_11	2017.Mor.4	18th c.	yellow/blue	haidate	brocade lining
4_13	2017.Mor.4	18th c.	blue	kote	embroidered lining
5_1	2017.Mor.5	after 1926	green	kusazuri	lacing braid
5_2	2017.Mor.5	after 1926	white	kusazuri	lacing braid
5_9	2017.Mor.5	after 1926	yellow	do	tassel
7_4	2017.Mor.7	17th c.	blue	kusazuri	lacing braid
7_8	2017.Mor.7	17th c.	light blue/yellow	kote	brocade lining
8_12	2017.Mor.8	17th c.	blue	kusazuri	lacing braid
8_18	2017.Mor.8	17th c.	yellow	sode	internal lining
8_4	2017.Mor.8	early 16th c.	blue	suneate	cord
8_7	2017.Mor.8	early 16th c.	light orange	kabuto	lacing braid
9_17	2017.Mor.9	late 19th c.	yellow	kabuto	tassel
9_5	2017.Mor.9	late 19th c.	orange	kusazuri	lacing braid
9_6	2017.Mor.9	late 19th c.	orange	kusazuri	lacing braid

c. is the abbreviation for century.

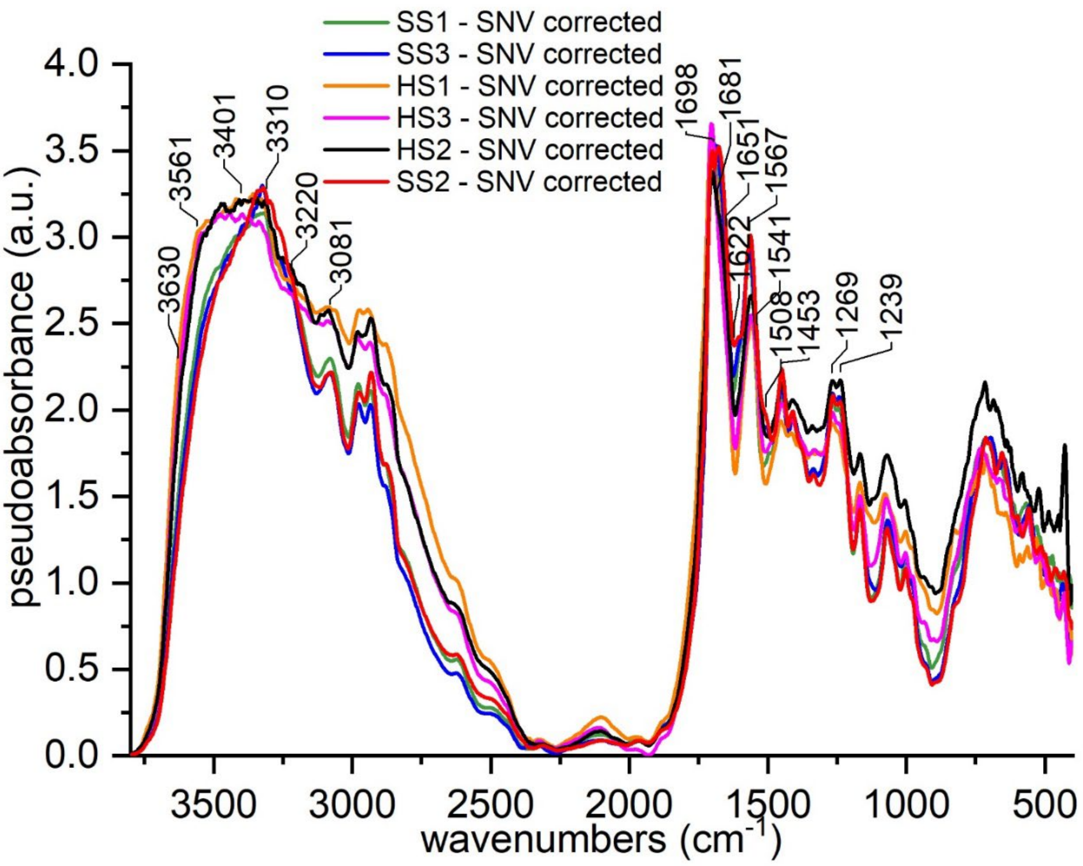

Figure S2. ER-FTIR spectra of hard and soft silk references (region 3800-400 cm^{-1})

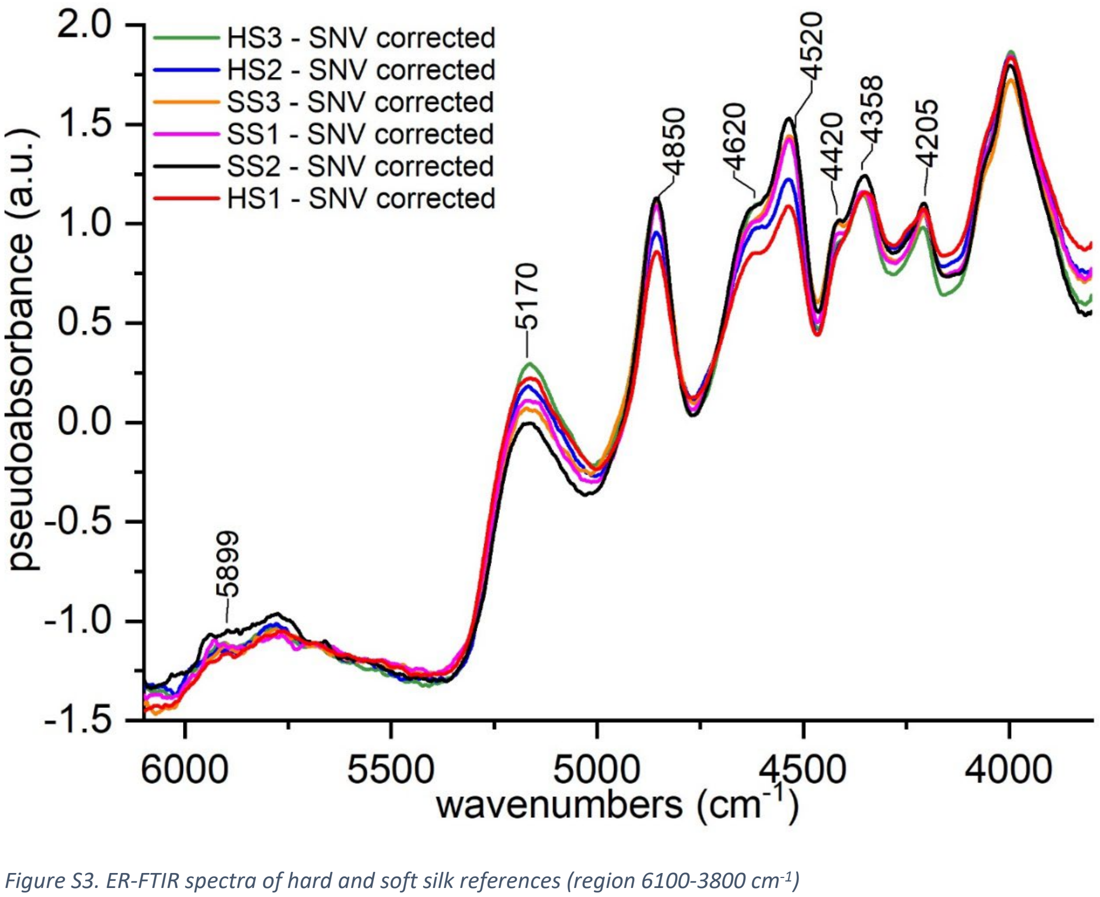

Figure S3. ER-FTIR spectra of hard and soft silk references (region 6100-3800 cm^{-1})
